# Ureterouterine fistula post caesarean section: a case report

**DOI:** 10.1186/1757-1626-1-253

**Published:** 2008-10-20

**Authors:** Katherine A Lanary, Hashim Hashim, John Iacovou

**Affiliations:** 1 Department of Urology, The Great Western Hospital, Marlborough Road, Swindon, Wiltshire, SN3 6BB, UK

## Abstract

**Background:**

Ureteric injury is usually iatrogenic in origin, in particular as a result of laparoscopic or gynaecological surgery. Of those possible complications of ureteric injury, uretero-uterine fistulae are a rarity. The most common presentation of uretero-uterine fistulae is in women who have had a caesarean section.

**Case:**

We present the case of a 35 year old woman who presented with continuous vaginal discharge three weeks after undergoing caesarean section.

**Conclusion:**

Management of ureterouterine fistulae aims to conserve renal function and restore ureteral integrity. The relative rareity of such fistulae however means that there are no clear guidelines on their management. The resolution of this case and general management of uretero-uterine fistulae are discussed.

## Case presentation

A 35 year old primiparous woman underwent an emergency caesarean section due to failure to progress in the second stage of labour. After a successful operative delivery, mother and child were discharged three days later, having had an uneventful recovery.

**Figure 1:**
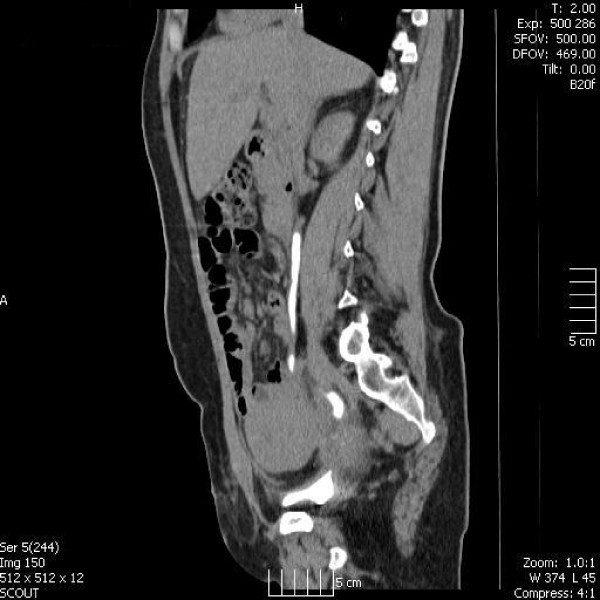
CT-IVU showing contrast in the right ureter draining into the uterus.

**Figure 2:**
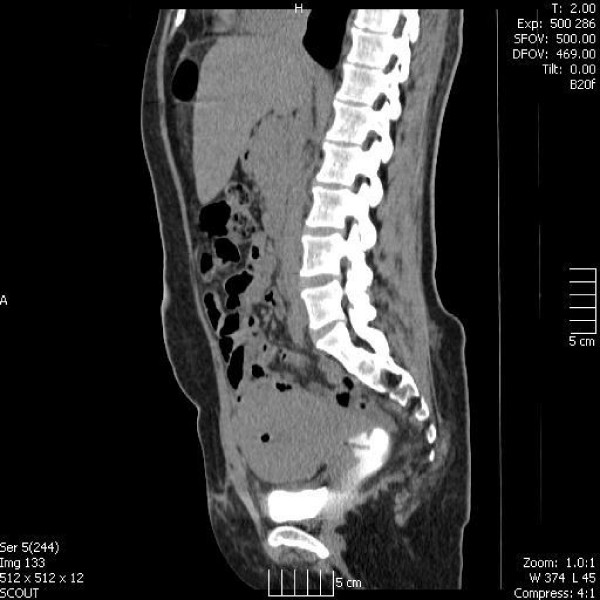
CT-IVU showing contrast in the right ureter draining into the uterus.

Eighteen days post caesarean section, the mother presented to hospital with continuous vaginal discharge necessitating pad changes every hour. The discharge was clear, consistent with urine. She underwent a computed tomography intravenous urogram (CT-IVU) which suggested a diagnosis of a uretero-uterine fistula (Figures 1 and 2).

**Figure 3:**
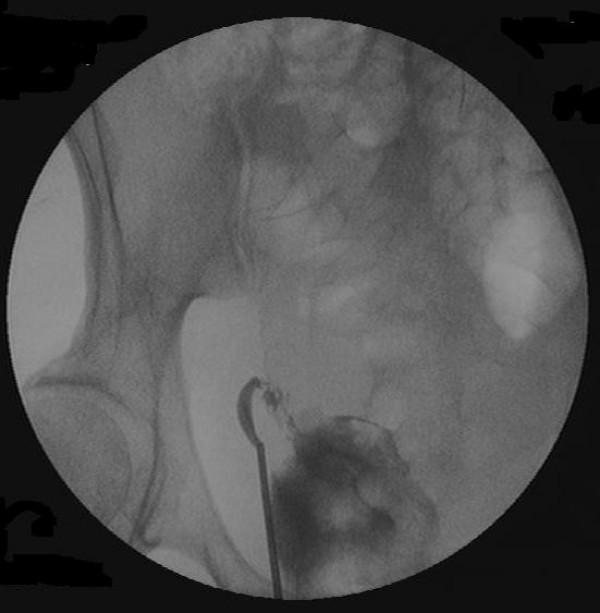
Retrograde ureterography showing the passage of contrast from the right ureter to the uterus.

Two days later, an examination under anaesthesia, and a cystoscopy and ureteroscopy, were performed. A small amount of blood was found on vaginal examination and cystoscopy revealed normal bladder and ureteric orifices. A guidewire inserted in the right ureter under imaging would only advance approximately 6 cm up the ureter. Retrograde ureterography was performed and showed the passage of dye from the right ureter to the uterus (Figure 3). The procedure was abandoned and a nephrostomy was inserted post-operatively to help divert urine away from the fistula.

**Figure 4:**
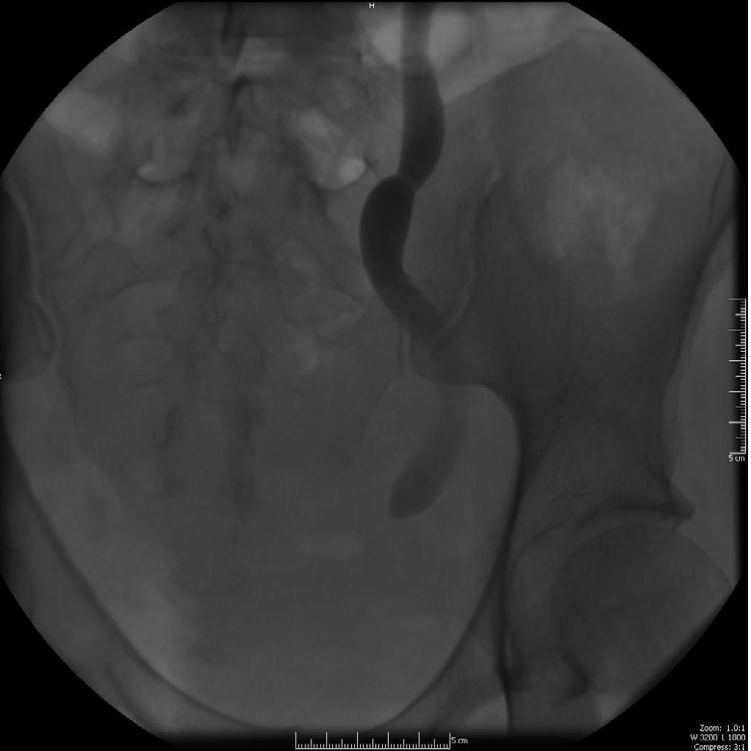
Nephrostogram showing complete obstruction of the right ureter.

After a two months interval, the mother returned to clinic for follow-up and reported that she had not experienced any further vaginal leakage of urine. In order to assess the condition of the ureter and fistula, a nephrostogram was performed which showed complete blockage of the ureter (Figure 4). Following the nephrostogram, vaginal discharge of urine recurred indicating persistence of the fistula.

**Figure 5:**
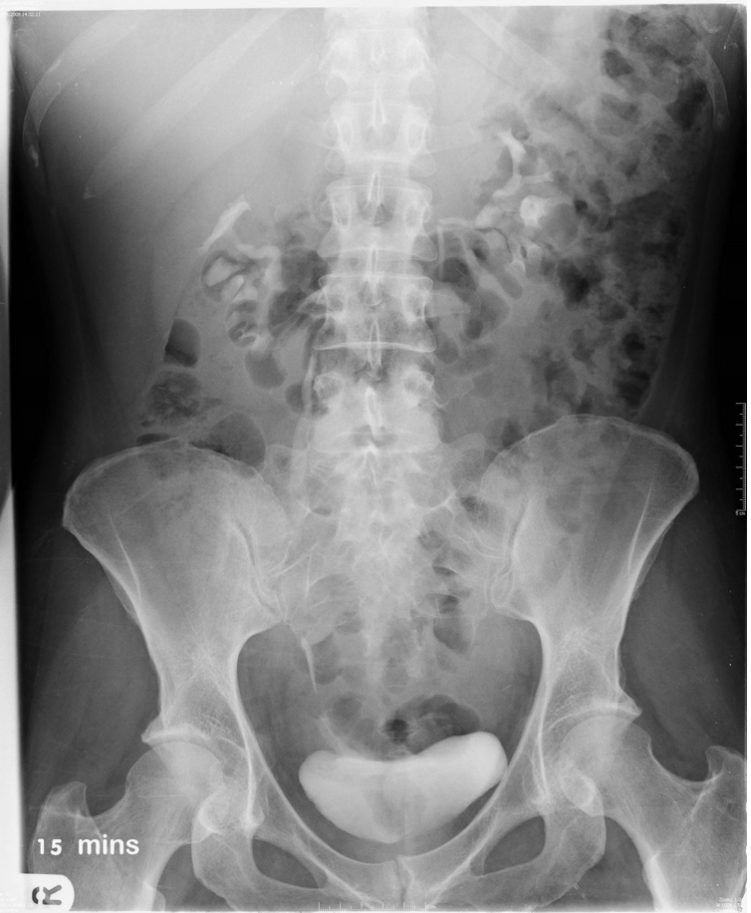
Post re-implantation intravenous urogram.

Six weeks later she underwent re-implantation of the right ureter and JJ-stent insertion. A suprapubic and urethral catheter were inserted and the nephrostomy clamped. The urethral catheter was removed five days post-operatively and a cystogram performed 10 days post-operatively showed no evidence of an anastomotic leak. The nephrostomy tube was removed under radiological guidance, and was inadvertently accompanied by the JJ-stent. One week later, an IVU revealed both right and left ureters to be draining well with no evidence of obstruction or fistula (Figure 5). The patient went on to make a full recovery.

## Discussion

Ureteric injuries are most commonly of iatrogenic origin, occurring as a result of intra-operative manoeuvres during abdominal or pelvic surgery. The incidence of ureteric injury during laparoscopic surgery has been cited as between 0.5 and 3% [1]. Uretero-uterine fistulae most commonly follow caesarean sections [2], where the rate of ureteric injury in the UK in 1997 was reported to be 0.27 cases per 1000 caesarean sections [3].

A relative rarity, uretero-uterine fistulae constitute less than 6% of all urinary tract fistulae [4]. Presentation of such fistulae is usually in the form of paradoxical incontinence as described by the patient above – that is, incontinence of urine in the presence of normal voiding.

The goals for management of uretero-uterine fistulae are the conservation of renal function and restoration of ureteral integrity through ureteroneo-cystostomy or end to end anastomosis. As in the case above, percutaneous nephrostomy may be used to divert urine and ensure adequate drainage thus conserving renal function and allowing any infection and inflammation to settle, with re-implantation of the ureter after an interval of about three months. Alternatively, primary surgery to re-implant the ureter may be used. In the absence of sepsis this has been suggested to be favourable in that it avoids the additional procedure of a nephrostomy and its associated risks [4], however this may be technically difficult post-delivery as the uterus would still be expanded making re-anastomosis or even re-implantation difficult.

There are no clear guidelines as to how to manage uretero-uterine fistulae as they are rare compared to other fistulae. There are also no clear operative techniques on managing such fistulae, for example. Should the ureter be dissected down to the bladder or should the ureter be transected above the fistula and re-implanted? Should the ureter be tunnelled or not? The long term effects of urine on the uterus are also poorly reported in the literature.

These questions are difficult to answer but some operative and post-operative principles include those adapted from ureteric trauma literature. These include using tension-free interrupted absorbable sutures to the ureter and bladder, placing anchoring sutures to the serosal surface of the ureter to secure it to the bladder, placing a stent across the anastomosis for six weeks. A non-suction drain should be placed at the site of the implant and removed after two days along with the urethral catheter. The urethral catheter can be removed the same day as the drain or a couple of days later. Antimuscarinic drugs may be required as having two balloons in the bladder can cause spasms. A cystogram will need to be performed at two weeks and if there is no leakage then the suprapubic catheter can be removed. At three months, an IVU, and possibly a MAG3 renogram will need to be obtained to confirm patency of the anastomosis [5]. The patient can then be discharged from follow-up if all the investigations are normal.

## Consent

Written informed consent was obtained from the patient for publication of this case report and accompanying images. A copy of the written consent is available for review by the Editor-in-Chief of this journal.

## Competing interests

The authors declare that they have no competing interests.

## Authors' contributions

The above investigations and treatment took place under the care of JI (Consultant Urologist) and HH (Specialist Registrar in Urology). KL (House Officer in Urology) was the main author of the manuscript with input from HH and JI. All authors read and approved the final manuscript.
